# Aetiology of long bone chronic osteomyelitis: an analysis of the current situation in one region in Egypt

**DOI:** 10.1007/s00590-022-03429-2

**Published:** 2022-11-21

**Authors:** Ahmed Elsheikh, Akram Hashish, Mai Kamal, Sayed El-Mohammadi, Yasser Ismael

**Affiliations:** 1 Orthopaedic Surgery Department, Faculty of Medicine, Benha University, Fareed Nada Street, Benha, 13511 Egypt; 2 Orthopaedic Surgery Department, Red Crescent Hospital, Cairo, Egypt; 3 Clinical Pathology Department, Faculty of Medicine, Benha University, Benha, Egypt

**Keywords:** Osteomyelitis, Gram-negative bacilli, Methicillin-resistant, *Staphylococcus aureus*, Polymicrobial, Multidrug-resistant

## Abstract

**Introduction:**

Chronic osteomyelitis (COM) is a devastating infection requiring a multidisciplinary approach, including radiology, microbiology, pathology, and orthopaedic surgery to treat. The present study analysed the bacterial profile causing chronic osteomyelitis and their antibiogram in our region.

**Patients and methods:**

This prospective study was done on a consecutive group of patients who underwent surgical debridement for long bone COM. Three to six deep tissue samples were collected during the index debridement for microbiology and one sample for histopathology. Antimicrobial sensitivity testing used an automated bacterial identification system. Gram stain was used to identify the bacteria type from its size, shape, and arrangement of bacterial growth.

**Results:**

Intra-operative deep tissue and bone specimens accurately identified causative bacteria in 84.8% of patients. Gram-ve bacilli (GNB) were the most common causative organisms in 51.6% of all growing samples (36.4% isolated G-ve and 15.2% mixed with G + ve). Thirty-three patients (30 males/three females) were included; the mean age at index debridement surgery was 37.1 years. Half of the cohort had no metalwork. The aetiology of COM was post-operative infection in half of the patients.

**Conclusion:**

There may be concerning features in our patients’ aetiologies and causative organisms; closed fractures turn into COM postoperatively, several unsuccessful attempts, delayed index debridement, and more GNB. Plans need to be applied to break the cycle and improve outcomes.

## Introduction

Osteomyelitis is characterized by low-grade inflammation caused by persistent pathogenic microorganisms with bone destruction and necrosis. It is still one of the most challenging conditions due to its long course, complex treatment, and risk of recurrence [1, 2]. Chronic osteomyelitis is intensely challenging to treat due to the poor blood supply, the devitalized tissues, the poor antibiotic penetration and the soft tissue envelope in some regions [1]. Biofilm-producing organisms adhere resiliently to the surface of the bone and implanted material, which makes them resistant to host defences and antibiotics [2, 3].

A definitive diagnosis of chronic osteomyelitis depends on identifying causative organisms through microbiological methods (culture and sensitivity) and histopathological examination of tissues [1]. Gram-positive organisms (specifically Staph aureus) remained the most commonly reported organisms to cause chronic osteomyelitis in long bones. However, other reports revealed paradoxical results with prevailing Gram-negative bacilli (GNB). Gram-negative osteomyelitis is hard to control and has increased multidrug resistance (MDR) and increased rates of recurrence [4–9].

This study aimed to analyse and discuss the microbiological results of intraoperatively collected samples of patients diagnosed with chronic osteomyelitis in one centre. With very few available published data from low resources regions [10], we planned to investigate the current situation to figure out the best way to control this debilitating disease and update our infection control measures.

## Patients and methods

### Study design

This prospective clinical study was done on a consecutive group of 33 patients who underwent surgical debridement for long bones chronic osteomyelitis in a single centre (Benha University Hospitals). The patients had their initial diagnosis and previous surgeries elsewhere in the region (Qalyubia governorate) and were referred to our centre later due to persistent infection. All patients had an established diagnosis of COM (clinical and radiological) in the appendicular system. Acute osteomyelitis or septic arthritis, periprosthetic infection, diabetic foot infection, or vertebral osteomyelitis were excluded from the study.

### Preoperative assessment

All patients included in the study were evaluated clinically, laboratory, and radiologically. Laboratory investigation included a complete blood picture, erythrocyte sedimentation rate, C-reactive protein, and other relevant preoperative investigations. A detailed history of previous surgeries and the metalwork used was also obtained.

Radiologically, anteroposterior and lateral plain X-rays were obtained to evaluate bone quality, sequestrated bone, sclerosis, the union of fractures, and loosening around metalwork. 18F-Fluorodeoxyglucose (FDG)-positron emission tomography (FDG PET-CT) scan was done on 70% of patients, aiming to provide a three-dimensional analysis of the infected area, helping the preoperative planning and determine the extent of resection during surgical debridement.

### Surgical debridement and samples collection

Surgical index debridement was done for all cases by the first author (AE) between February 2019 and February 2022. The extent of debridement was planned preoperatively. Antibiotic therapy was stopped 14 days before the index debridement surgery, and no routine antibiotic prophylaxis was given until bone/tissue biopsies were collected. Three to six deep bone/tissue samples were collected in leak-proof sterile containers using different instruments and immediately transferred to the Microbiology department [11].

### Microbiological examination

Standard bacteriological techniques were applied to isolate and identify the causative organisms. Specimens were cultured on primary culture media (nutrient, blood, and MacConkey agars), incubated at 37 °C for 24–48 h. Liquid samples were inoculated in blood culture broths incubated in Bact/alert system. Positive vials were cultured on the previous culture media. Colonial growth on the agar surface was inspected for colonial morphology, haemolysis on blood agar, swarming, or any other characteristic odour or pigment. Where mixed bacterial growth was observed, subculture was done. No molecular typing or PCR was performed.

Antimicrobial sensitivity testing used an automated bacterial identification system (Vitek 2 and Phoenix) that analyses MIC patterns and detects phenotypes for most tested organisms. Smear from the bacterial growth was prepared and fixed with one or two drops of methanol, stained with Gram stain and examined microscopically.

### Histopathological examination

One bone/soft tissue sample was sent for histopathological examination; the sample was collected and transferred immediately with no preservative fluid. The histopathologists were not aware of the microbiological results of the patients. The characteristic histopathological findings of COM are the persistence of dilated blood vessels, granulation tissue, inflammatory cell infiltration, devitalized bone, reactive new bone formation, and suppuration. A definitive diagnosis of COM was made based on deep tissue surgical cultures and histopathological analysis.

### Analysis of the results and statistical methods

Data management and statistical analysis were done using SPSS version 28 (IBM, Armonk, New York, USA). Quantitative data were assessed for normality using the Shapiro–Wilk test and direct data visualization methods. According to normality testing, numerical data were summarized as means and standard deviations or medians and ranges. Categorical data were summarized as numbers and percentages. The microbiology, histopathology, and culture results were plotted together to conclude and investigate the causative organisms and antibiotic culture and sensitivity.

## Results

This study was conducted on 33 patients (30 males and three females) suffering from chronic osteomyelitis (COM). There were 18 smokers, one EX smoker, and 14 nonsmokers. The mean age at the index debridement surgery was 37.1 years (range 7–73). Patient characteristics are detailed in Table 1.

**Table 1:** Patient characteristics

	Number	Percentage
*Cause of trauma in the studied patients*		
Road traffic accident	23	69.7
Direct trauma	3	9.1
Spontaneous infection (No trauma)	3	9.1
Falling from height	2	6.1
Gunshot	1	3.0
Post-compartment syndrome	1	3.0
*Bony status*		
Infected non-union	24	72.7
Infected united	6	18.2
Intact bone	3	9.1
*Local examination*		
Inflamed skin/draining sinus	31	93.9
Skin defect	3	9.1
*Bone affected*		
Tibia	16	48.5
Femur	10	30.3
Fibula	4	12.1
Ulna	2	6.1
Calcaneus	1	3.0
*Source of infection*		
Post-operative	17	51.5
Open fracture	13	39.4
Haematogenous	2	6.1
Primary chronic sclerosing OM	1	3.0
*Cierny-Mader classification*		
Diffuse OM	23	69.7
localized OM	6	18.2
Intramedullary OM	4	12.1
*Metalwork at time of index debridement*		
No implant	16	48.5
Plate	8	24.2
Nail	7	21.2
LRS	1	3.0
Ilizarov	1	3.0

	Median	Range
The interval between injury (in patients with fractures) and the onset of Infection	8.9 weeks	1–59
Infection duration (from date of onset of infection till the index debridement surgery)	23.4 months	1–75
Operations done elsewhere to control/eradicate infection	3	0–7

### Laboratory and culture findings

The mean WBCs were 7.1 ± 1.1. The mean ESR was 48.8 ± 12.2. The mean CRP was 22.3 ± 9.7. The mean number of samples collected for microbiology was 5 ± 1. Growth was reported in 28 patients (84.8%). GNB were the most common causative organisms in 51.5% of the cohort, slightly higher than Gram-positive (48.5%). Looking at the spectrum of organisms, cultures grew isolated GNB in 36.4%, gram-positive in 33.3%, mixed in 15.2%, and no growth in 15.2%. Monomicrobial infection was found in 18 patients (54.5%), while polymicrobial infection (different identified organisms) was evident in a third of the cohort (ten patients, 30.3%) (Table 2).

**Table 2:** Culture characteristics in the studied patients

*Culture results*	
Growth	28 (84.8%)
No growth	5 (15.2)
Organisms of the whole cohort*	
Gram-negative	17 (51.5%)
Gram-positive	16 (48.5%)
Culture results**	
Isolated gram-negative	12 (36.4)
Isolated gram-positive	11 (33.3)
Mixed (gram-positive and negative)	5 (15.2)
No growth	5 (15.2)
Monomicrobial versus polymicrobial**	
Monomicrobial	18 (54.5%)
Polymicrobial	10 (30.3%)
No growth	5 (15.2)

*Percentage of all grown samples (overlap due to mixed growth)

**Percentage of all cohort patients

### Organism identity and sensitivity

MRSA was the most frequently identified single organism to grow in all cultures (isolated or mixed) (36.4%). Klebsiella was the most frequent single GNB (15.2%); However, there were some results of Gram-negative cultures (12.1%) that could not identify the organism specifically (Table 3). MDR Klebsiella (*n* = 5) was only 20–40% sensitive to gentamycin, amikacin, ciprofloxacin, and trimethoprim–sulphamethoxazole. Pseudomonas (*n* = 3) was 100% sensitive towards piperacillin/tazobactam, gentamycin, amikacin, ciprofloxacin, and levofloxacin and 66% sensitive to cefipime and imipenem. *E. Coli* (*n* = 3) was sensitive to piperacillin–tazobactam, ciprofloxacin, and levofloxacin in 66.7% and sensitive to amoxicillin–clavulanic acid and trimethoprim–sulphamethoxazole in only 33.3%. *Proteus* (*n *= 3) was 67.3% sensitive to piperacillin/tazobactam, meropenem, ceftazidime, and amikacin.

**Table 3:** Detailed culture results in the studied patients

	*N*	%*
*Gram-positive organism*		
MRSA	12	36.4
MSSA	2	6.1
Enterococci	2	6.1
Coagulase negative staph	1	3.0
*Gram-negative organism*		
*Klebsiella*	5	15.2
Non-specific G-ve bacilli	4	12.1
*Pseudomonas*	3	9.1
*Ecoli*	3	9.1
*Proteus*	3	9.1
*Stenotrophomonas*	1	3.0
*Acinetobacter baumannii*	1	3.0
*Morganella morganii*	1	3.0
Aeromonas hydrophila caviae	1	3.0

*Percentage of all positive growth samples (*n* = 28)

## Discussion

In this study, intra-operative deep tissue and bone specimens accurately identified causative bacteria in 84.8% of patients. All cases underwent index debridement and sample collection by the same surgeon, with three to six collected specimens. There was an increased incidence of G-negative osteomyelitis in 51.5% of the whole cohort, either as isolated GNB (36.4%) or mixed gram-positive and negative (15.2%).

Chronic osteomyelitis is a challenge for orthopaedic surgeons. Appropriate diagnosis and treatment of chronic osteomyelitis require microbiologic cultures of the infected bone and proper antibiotics according to culture and sensitivity. Bone provides a unique harbour for microorganisms that produce biofilms, allowing them to attach resiliently to biologic and implanted surfaces, remaining insusceptible to host defences and antibiotics [2].

Several studies analysed the bacteriological profile in long bone osteomyelitis [3, 5–8, 12–16]. Reports from low resources settings and developing countries are sparse [4, 9, 10, 17, 18]. Nevertheless, other studies investigated the aetiology and bacteriology of COM in a diabetic foot or joint replacement infection or mixed group of patients [19–21]. We did not reflect on these studies due to the particular pathology in each situation which is different from implant-related long bone osteomyelitis.

Sheehy et al. and Dudareva et al. reported 66% and 67.7% culture-positive results, respectively, compared to 84.4% in this study [12, 15]. Sheehy et al. reported a range of samples from 0 to 10, with no samples analysed in ten patients out of 166 (6%). Marais et al. [7]. recorded higher culture-positive results (90.9%); however, this result represents only 11 of 26 patients who underwent surgical debridement and intra-operative bone biopsies.

The lack of growth in the collected samples of any study has some explanations. Antibiotics should be stopped for 14 days in clinically stable patients before surgery. Samples should be collected from the deep bone and soft tissues. Multiple samples should be collected with different instruments. Proper containers and a timely chain of transfer should be applied. However, we applied all these precautions in this study, with 15% of samples failing to grow organisms [11].

Other factors are related to microbiological methods and techniques. Pretreatment of samples with specific chemicals could release the organism from the biofilm. Also, physical methods like sonication offer similar effects. Extended cultures also provide extra opportunities to grow weak organisms [22]. This study could not apply such methods due to unavailability or logistic problems.

Although sinus tract swabs are still one of the diagnostic tools of COM and may provide the accurate causative organism, tissue specimen remains the gold standard for diagnosing COM and providing accurate causative organisms. Vemu et al. compared tissue specimens and sinus tract swabs; positive culture in tissue specimens was 60.7% compared to 37.7% in sinus swabs [23]. Contamination with nonpathogenic bacteria or other naturally occurring random organisms during the sampling process could explain this outcome.

The current study showed that chronic osteomyelitis was the highest in middle age (median 37y), which agrees with other studies [4, 7, 15, 23]. Road traffic accidents frequently occur among those age groups; such high-energy trauma often leads to open fractures and soft tissue trauma and potential risk factors for traumatic osteomyelitis. In the current study, the male/female ratio was 10:1, which is very similar to previous studies [7, 12, 13, 15, 23].

The aetiology of COM in our study was post-operative in half of the patients. In other studies, haematogenous osteomyelitis or OM following open fractures were the leading cause [13, 15, 16]. Post-operative COM was only 29.3%, 9.9%, and 2.8% of the whole cohorts, respectively [4, 12, 13]. This finding is very critical, we had difficulty analysing this high rate, but there were some clues. Patients were operated on previously in different hospitals. Also, there is no universal MRSA screen or urine dipstick screening. We could not document the general health status or the soft tissue injury. Additionally, antibiotic and follow-up regimens were different, with a tendency to give strong antibiotics for a long time and to do daily dressing on the post-operative wounds. Finally, we do not have the number of surgeries at each hospital to get an actual incidence of surgical site infection.

In the current study, the tibia (48.5%) was the most frequent site of chronic osteomyelitis. Many studies had similar findings [4, 7, 12, 13, 15, 16]. Two other studies reported the femur as the most frequent infected bone [14, 23]; in our report, the femur was the second affected bone (30.3%).

GNB organisms were the most common cultured bacteria in 51.5% of patients (alone or mixed with Gram + ve organisms). In the literature, GNB bacteria were prevalent in a few studies. GNB incidence has evolved; a historical paper by Meyers et al. reported a 28% incidence of GNB in 1973, increasing from 15% only in 1966 [8]. Recently, Marais et al. reported 26 patients in South Africa with COM in two groups: palliative versus curative. Their cohort had nearly equal numbers of haematogenous, post-operative and open fractures, and the tibia was the most commonly affected bone. GNB infection was recorded in 61.5% (*n* = 8) compared to 38.5% (*n* = 5) Gram + ve [7].

In Egypt, two studies analysed the bacteriological profile in their centres within the context of reporting the surgical outcomes of single-stage debridement [17, 18]. El-Moatassem examined 16 patients with tibial COM, who grew Gram + ve bacteria (MSSA and MRSA) in 12 (75%) and Pseudomonas in one; three more did not grow any organism [18]. Badie and Arafa reported the results of 30 cases with long bone COM; half had tibial involvement. They had 56% haematogenous, 20% post-operative infection, and the rest were open fractures. Cultures were positive in 93% of cases, and GNB grew in 32% [17]. Both studies emphasized the surgical aspects; we could not retrieve further details on sensitivity or resistance patterns.

Agaja and Ayorinde reported similar trends in their cohort of 107 patients from Nigeria, with GNB (43%) slightly higher than Gram + ve (37.4%). However, 81.3% of their patients had haematogenous osteomyelitis [4]. Mousa et al. studied 134 patients from Iraq in four groups: haematogenous, exogenous (open fractures), post-operative, and mastoiditis. GNB (Pseudomonas species and Klebsiella species) were prevalent in the exogenous and post-operative osteomyelitis groups [9].

Carvalho et al. published a study that included 101 patients from Brazil who presented with isolated GNB osteomyelitis; 43% COM and 32% were associated with open fractures, and the remaining were post-operative. Clinical remission was achieved in 60% of patients. Enterobacter and *Acinetobacter baumannii* were the commonest. Pseudomonas came third, with sensitivity ranging from 70 to 80% towards the GNB targeting antibiotics [5]. Jorge et al. investigated 193 patients diagnosed with osteomyelitis following fractures (closed or open) in Brazil. They reported GNB in 51.8% of patients. *Pseudomonas aeruginosa* and *A. baumannii* were the most frequent [14].

Koutserimpas et al. analysed 14 patients with MDR and extended drug resistance (XDR) in one centre in Greece over ten years; most had post-operative osteomyelitis. *Acinetobacter* *baumannii* was the most frequent, followed by *Escherichia coli* and *Klebsiella pneumoniae*. They highlighted that *A. baumanii* was repeatedly a leading cause of hospital-acquired infection in their region [6]. In our cohort, Klebsiella was the commonest, followed by *Pseudomonas*, *Proteus* and *Ecoli*. *Acinetobacter* was found in one patient with no Enterobacter.

In the present study, MRSA remains the most frequent single isolate at 36.4% (*n* = 12), in agreement with many previous studies, which reported an incidence ranging between 20 and 48% [4, 12, 13, 15, 16, 23]. However, two studies from the same team ten years apart [12, 15] reported very similar incidences of staphylococcus infections (31.3%–37.5%) in both cohorts, yet, there was a declining proportion of MRSA from 30.8 to 11.4% of all staph infections, respectively**.** Strict applied hospital infection prevention policies could have resulted in this reduction, including preoperative MRSA screening and decolonization with topical nasal mupirocin and chlorhexidine shower.

In the current study, 85.7% of S. aureus strains were resistant to methicillin (MRSA). In addition, all MRSA were 100% sensitive to amikacin, ciprofloxacin, erythromycin, linezolid, vancomycin, and clindamycin. This spectrum of sensitivities draws attention to the current problem and should attract more practical steps to reduce MRSA osteomyelitis. GNB sensitivity profile also represents a challenge; GNB is more likely to be resistant to multiple drugs, e.g. *Klebsiella*, which is 40% sensitive only to gentamicin and amikacin and 20% sensitive to ciprofloxacin and sulphamethoxazole.

In this study, we aimed to raise voices about the differences in causes and bacteriological profiles of COM in low resources settings. The management of chronic osteomyelitis in low resources countries had more obstacles compared to the developed world [10]. However, the high-quality literature about the bacteriological spectrum and sensitivities profile was driven by developed countries [12, 15]. Consensus and recommendations for antibiotic management were also reported from specialized centres in high-income countries [24, 25]. The contradictory findings in this study would change the current practice of choice of antibiotic prophylaxis and treatment in low resources settings.

## Limitations

This study has some limitations due to the relatively small sample size, heterogeneity of cases and single hospital location. The lack of data about the previous surgeries in different hospitals made tracing challenging. We could not identify the causes behind the increased post-operative infection which is a major preventable source in our cohort. More work needs to be done to analyse the GNB endemic and how to fight it.

## Conclusion

There may be a shift in our region’s aetiologies and causative organisms; closed fractures turn into COM postoperatively, several unsuccessful attempts, delayed index debridement, and more GNB organisms. Plans need to be applied to break the cycle and improve outcomes.
